# Light-Curable Methacrylated Carboxymethyl Chitosan Hydrogel Incorporating Bone Morphogenic Protein-2-Immobilized Bioactive Glass for Spinal Bone Regeneration

**DOI:** 10.34133/bmr.0391

**Published:** 2026-07-07

**Authors:** Mi Yeon Ha, Gun-Jae Jeong, Jae Taek Hong, Hye June Byun, Dae Hyeok Yang, Ju Woong Jang, Heung Jae Chun

**Affiliations:** ^1^ Institute of Cell and Tissue Engineering, College of Medicine, The Catholic University of Korea, Seoul 06591, Republic of Korea.; ^2^ School of Nanomedical Engineering, Korea National University of Transportation, Chungju-si, Chungcheongbuk-do 27469, Republic of Korea.; ^3^ Department of Neurosurgery, Eunpyeong St. Mary’s Hospital, College of Medicine, The Catholic University of Korea, Seoul 03312, Republic of Korea.; ^4^ Department of Medical Sciences, College of Medicine, The Catholic University of Korea, Seoul 06591, Republic of Korea.; ^5^ Renew Medical Co. Ltd., Bucheon-si, Gyeonggi-do 14532, Republic of Korea.; ^6^ Department of Medical Life Sciences, College of Medicine, The Catholic University of Korea, Seoul 06591, Republic of Korea.

## Abstract

Bioactive glass (BG) has emerged as a promising material for bone tissue engineering due to its ability to release osteogenic ions and establish robust interfaces with living tissues, thereby contributing to spinal bone regeneration by facilitating osteointegration and structural stability. Despite these advantageous properties, reconstructing spinal bone defects remains particularly challenging because BG is typically available in powder form, which lacks sufficient mechanical strength and is difficult to handle surgically, especially given the substantial mechanical demands of the spinal column. To overcome these limitations, we employed an injectable, light-curable methacrylated carboxymethyl chitosan (CMCSMA) hydrogel to enable minimally invasive delivery and precise defect filling. Furthermore, to complement BG’s strong osteoconductive capacity with osteoinductive functionality, bone morphogenetic protein-2 (BMP-2) was immobilized onto BG particles (BG/BMP-2), creating a composite scaffold with dual biological activity. The BG/BMP-2/hydrogel system demonstrated sustained BMP-2 release, enhanced osteogenesis, and substantial new bone formation, underscoring the synergy between BG’s bioactive ion release and hydroxyapatite-forming ability. The photocurable hydrogel matrix provided conformal adaptation to defect geometry, potentially improving surgical outcomes. Collectively, this composite represents a promising strategy for minimally invasive and mechanically resilient spinal bone regeneration.

## Introduction

Bioactive glass (BG) has been extensively used in tissue regeneration because of its remarkable properties such as biocompatibility, biodegradability, osteogenic potential, and angiogenic capability [1]. These features enable BG to support cellular adhesion, proliferation, and differentiation, thereby promoting bone formation and vascularization [2,3]. Furthermore, the degradation of BG under physiological conditions, accompanied by the release of bioactive ions, substantially contributes to tissue repair and regeneration. Consequently, BG has emerged as a highly attractive biomaterial for diverse biomedical applications, particularly in bone tissue engineering [4–6].

Despite recent advancements in bone tissue engineering, spinal bone defects and loss remain clinical challenges. Spinal bone loss can result from osteoporosis, traumatic injuries, tumors, infections, and degenerative conditions, all of which compromise the structural integrity of the vertebral column [7]. Because the spine bears substantial mechanical loads and protects critical neural elements, inadequate treatment of spinal defects can lead to chronic pain, instability, and neurological complications. Additionally, conventional bone grafting techniques such as autografts and allografts present several drawbacks, including donor site morbidity, potential immune reactions, and inconsistent fusion outcomes [8]. Therefore, there is an increasing need to explore innovative strategies that provide mechanical support, accelerate osteointegration, and promote robust bone regeneration in spinal environments.

In this context, the involvement of BG in bone healing has garnered considerable interest, primarily due to its dissolution behavior and the release of bioactive ions [9,10]. Upon implantation, BG releases calcium, phosphate, and silicon ions that stimulate cellular responses, induce osteoblastic activity, and accelerate the formation of a hydroxyl carbonated apatite (HCA) layer, a key component of natural bone mineral [1,11]. The presence of this HCA layer promotes osteointegration by establishing a strong bioactive interface with the host bone tissue, which represents a key mechanism of osteoconduction [12].

An additional strategy to augment the osteogenic potential of BG involves incorporating bone morphogenetic protein-2 (BMP-2), a well-known osteoinductive growth factor that not only stimulates osteoblast differentiation and extracellular matrix deposition [11], but also recruits mesenchymal stem cells and osteoprogenitor cells to defect sites. Through this osteoinduction, BMP-2 modulates key cellular processes, including adhesion, proliferation, migration, and differentiation, and acts synergistically with BG to promote robust bone regeneration, particularly in challenging anatomical sites such as the spine.

Hydrogels have gained prominence as scaffolding materials in tissue engineering due to their minimally invasive application and adaptability to complex defect sites [13–15]. They can encapsulate bioactive molecules and support cell–material interactions within a 3-dimensional (3D) matrix, making them especially effective in promoting tissue repair [13–16]. Among various hydrogel systems, those derived from chitosan are particularly advantageous because of their biocompatibility, biodegradability, inherent antibacterial properties, and chemical versatility for functional modification. Specifically, in situ-forming, light-curable hydrogels represent a promising approach for spinal repair, as they can be delivered to the defect site in liquid form and subsequently polymerized under controlled light exposure [17,18]. This feature offers several benefits, including conformability to irregularly shaped defects, ensuring intimate contact with host tissue, on-demand gelation, which reduces surgery time and enhances procedural control, and customization for the prolonged release of osteogenic cue, such as BMP-2, directly at the injury site. Collectively, these advantages make light-curable hydrogels a highly attractive option for delivering both BG particles and growth factors, providing structural support that compensates for the inherent limitations of BG powder while preserving mechanical stability and accelerating regenerative processes.

**Fig. 1.**
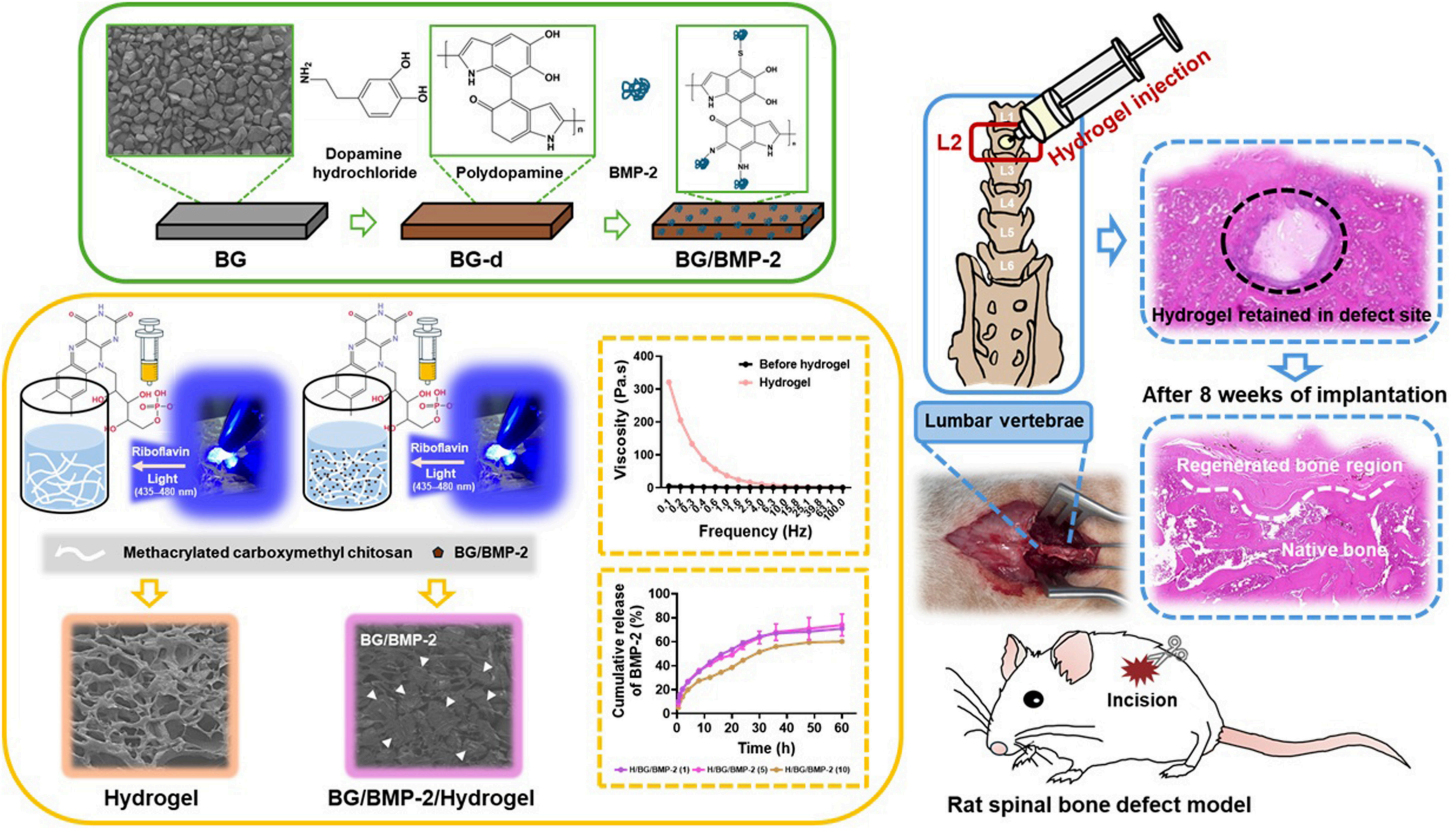
Scheme of in vitro and in vivo experimental design. Schematic of the experimental design for spinal bone regeneration, highlighting enhanced cell proliferation and regulated osteogenic differentiation by mixing BG and BG/BMP-2 into light-curable CMCSMA hydrogel.

We postulated that combining the complementary mechanisms of BG and BMP-2 would generate a synergistic effect, as BG provides a favorable osteoconductive and osteointegrative scaffold while BMP-2 supplies potent osteoinductive signaling to drive cellular differentiation and stem cell recruitment. Accordingly, our central hypothesis was that surface functionalization of BG with BMP-2 and its incorporation into a light-curable methacrylated carboxymethyl chitosan (CMCSMA) hydrogel could accelerate bone formation and enhance osseointegration in spinal defect models (Fig. 1). Based on this rationale, the present study aimed to evaluate BMP-2-functionalized BG/hydrogel composites for spinal bone regeneration through both in vitro and in vivo experiments, assessing osteogenic capacity, mechanical stability, and overall biocompatibility. Our findings offer valuable insights into a promising approach to address the critical clinical need for more effective spinal repair strategies, potentially improving patient outcomes and reducing complications associated with spinal bone defects.

## Materials and Methods

### Materials

Reagents including ioactive glass (BG; 45S, Purity ≥ 98%), dopamine hydrochloride (molecular weight [MW] = 189.64 g/mol, purity ≥ 95%), and glycidyl methacrylate (MW = 142.15 g/mol) were purchased from Sigma (USA). BMP-2 solution (10 μg/ml, 10 mM Tris-HCl, pH 8.5) was provided by Cellumed Co., Ltd. Simulated body fluid (SBF; S2028) was purchased from BIOSESANG (Republic of Korea). Carboxymethyl chitosan (CMCS; MW = 50 to 100 kDa, deacetylation degree ≥ 90%, degree of substitution [DS] ≥ 0.8) and riboflavin (MW = 478.33 g/mol, purity 98.3%) were purchased from Santa Cruz Biotechnology (USA). Dialysis tube (cutoff: 25 kDa) was supplied by Spectrum Laboratories Inc. (USA). Human bone marrow-derived mesenchymal stem cells (BMSCs) of passage 2 were purchased from LONZA (Switzerland). All chemicals were used as received without further purification.

### Coating of BG particles with PDA-mediated BMP-2

To prepare polydopamine (PDA)-coated BG, 100 mg of BG particles was dispersed in a dopamine hydrochloride solution (2 mg/ml, 10 mM Tris-HCl, pH 8.5) and agitated on an orbital shaker at 50 rpm for 4 h. To remove residual unreacted dopamine, the mixture was subsequently washed with distilled water (DW) under continuous stirring overnight at room temperature (RT). Next, 5 mg of the resulting PDA-coated BG was immersed in a BMP-2 solution (10 μg/ml, 10 mM Tris-HCl, pH 8.5) and maintained at 37 °C overnight. Following incubation, the BG/BMP-2 particles were washed with DW, while the remaining supernatant was collected to quantify the unbound protein. The immobilization efficiency of BMP-2 was evaluated indirectly via an enzyme-linked immunosorbent assay (ELISA). Specifically, the BMP-2 concentration in the collected supernatant was determined using a commercial ELISA kit (DBP200, R&D Systems, USA). The total mass of immobilized BMP-2 was then calculated by subtracting the BMP-2 content detected in the supernatant from the initial amount present in 500 μl of original working solution.

### Characterization of BMP-2-modified BG (BG/BMP-2)

Surface elemental composition and chemical states of the functionalized BG/BMP-2 composites were analyzed at RT utilizing x-ray photoelectron spectroscopy (XPS; Nexas, Thermo Fisher Scientific, USA). The XPS measurements were conducted with pass energies set to 200 eV for survey scans and 50 eV for high-resolution spectra. Additionally, the morphological characteristics and particle size distributions of both pristine BG and BG/BMP-2 were evaluated using a scanning electron microscope (SEM; SU8220, Hitachi, Japan). To prevent sample charging during SEM observation, all specimens were sputter-coated with a thin layer of platinum at RT prior to imaging at multiple magnifications.

### In vitro investigation of material bioactivity

The in vitro bioactivity of the BG and BG/BMP-2 composites was assessed by monitoring the formation of a biomimetic hydroxyapatite layer after immersion in SBF, which is a standard procedure for evaluating bone-bonding ability. Briefly, 20 mg of each sample was soaked in 20 ml of SBF and maintained at 37 °C for periods of 1 and 7 d. Following the designated incubation times, the materials were carefully rinsed with DW and allowed to air-dry. The surface topography of the dried specimens was then examined via SEM (SU8220, Hitachi, Japan). Prior to observation at an accelerating voltage of 2 kV, the samples were coated with platinum for 1 min using a sputter coater (SMC12R-Plus, Semian, Republic of Korea) [12].

### Preparation of CMCSMA hydrogel

For the synthesis of CMCSMA, 1 g of CMCS was first dissolved in DW to yield a 1% (w/v) solution. The pH of this solution was adjusted to 9.0 using 0.1 N NaOH, after which 500 μl of glycidyl methacrylate was added dropwise under continuous stirring. This reaction mixture was stirred at RT for 24 h. To halt the reaction, the pH was neutralized to 7.0 using 0.1 N HCl. The resulting mixture was purified against DW for 7 d via dialysis (25 kDa MWCO membrane), with the dialysate refreshed daily to remove unreacted precursors. The purified solution was then lyophilized at –70 °C for 7 d to produce solid CMCSMA powder, which was subsequently sterilized using ethylene oxide (EO) gas. To prepare the hydrogels, the sterilized powder was completely dissolved in phosphate-buffered saline (PBS) to form a 7% (w/v) precursor solution. Following thorough mixing, free-radical photopolymerization of the methacrylate groups was initiated by adding riboflavin (120 μM) as a photoinitiator. Gelation was achieved by exposing the mixture to blue light (435 to 480 nm) for 60 s utilizing a bioresin curing device (FP SQUARED, Republic of Korea) [19].

### ^1^H NMR spectroscopy analysis

The successful grafting and the degree of methacrylation on the CMCS backbone were verified via ^1^H nuclear magnetic resonance (NMR) spectroscopy. Briefly, 0.1% (w/v) solutions of CMCS and CMCSMA powders were prepared by dissolving each sample in deuterated water (D_2_O) under vortex mixing. The ^1^H NMR spectra were recorded using a 400-MHz spectrometer (Bruker ULTRASHIELD 300; Bruker, USA).

### Measurement of mechanical strength

To evaluate the compressive mechanical properties, various hydrogel composites were prepared. The base hydrogel matrix was fabricated by blending the CMCSMA solution with the riboflavin photoinitiator and curing it under blue light. For the composite groups, either pristine BG or BMP-2-immobilized BG particles were uniformly dispersed into the CMCSMA precursor solution at varying concentrations of 1%, 5%, and 10% (w/v) prior to photopolymerization. These were designated as H/BG (1, 5, 10) and H/BG/BMP-2 (1, 5, 10), respectively. The compressive strength of cylindrical hydrogel specimens (0.7 mm diameter, 0.5 mm thickness) was tested at RT using a Universal Testing Machine (INSTRON, USA) equipped with a 1-kN load cell. The tests were performed at a compression rate of 5 mm/min up to a maximum strain of 80%, under controlled environmental conditions (25 ± 1 °C under a relative humidity of 50% ± 5%). Each formulation was tested in triplicate. Real-time force and displacement data were recorded to plot stress–strain curves, from which compressive stress (force per unit initial cross-sectional area) and strain (change in height over initial thickness) were derived.

### Rheological measurements

The gelation properties of the hydrogel formulations (7% in PBS) were assessed through rheological measurements on a rheometer (TA Instruments, USA) fitted with a 20-mm parallel plate and a gap of 0.1 mm. Storage (*G*′) and loss (*G*″) modulus were measured in frequency sweep mode ranging from 1 to 100 rad/s at a constant strain of 1% at 25 °C.

### Measurement of pH variation

To monitor changes in the local pH and assess ionic exchange, the fabricated samples were incubated in 10 ml of SBF at 37 °C without agitation. The pH of the surrounding media was recorded at predetermined intervals (0, 2, 4, 8, 12, 24, 48, and 72 h) utilizing a calibrated digital pH meter (Ohaus, USA). To guarantee solution uniformity, all vials were mildly agitated prior to each measurement.

### Cumulative BMP-2 release test

To characterize the BMP-2 release pattern, the H/BG/BMP-2 groups were placed in 3 ml of PBS in a shaking incubator at 37 °C and 100 rpm. At time points up to 60 h, the release solutions were withdrawn and replaced with fresh PBS, and the released protein concentration was determined using a BMP-2 ELIZA kit (DBP200, R&D Systems, USA) according to the manufacturer’s instructions. ELISA was also used to quantify BMP-2 in 500 μl of the original solution, and the released BMP-2 was calculated by subtracting the supernatant concentration from that of the original solution. Experiments were conducted according to the manufacturer’s protocol, with sample absorbance recorded on an ELISA reader at 450 nm (Power wave XS, BIO-TEK, USA) [20].

### Swelling ratio of the hydrogel

The scaffolds were fabricated in the same shape (to fit 24-well plates) and volume (1 ml). Swelling behavior was assessed in PBS at 37 °C with constant shaking at 100 rpm. The dry weight of each sample was recorded. The scaffolds were thereafter incubated in PBS at 37 °C, and their wet weights were obtained at various time intervals (0, 4, 8, 12, 16, 24, and 30 h). The swelling ratio was calculated using the following equation, where *W*_W_ represents the wet weight of the sample and *W*_i_ denotes its initial dry weight [21].

$$\text{Swelling ratio}\;\left(\%\right)=\left(W_{W}/W_{i}\right)\times 100$$ (1)

### Cell culture

Human BMSCs acquired from LONZA (Basel, Switzerland) were utilized for the in vitro assays. The cells were propagated in MSCBM medium (LONZA) in a humidified incubator at 37 °C with 5% CO₂. The culture medium was replenished every third day. Once the cells reached approximately 80% to 90% confluence, they were detached with 0.25% trypsin-EDTA (Gibco, MA, USA) and subcultured. Cells between passages 2 and 6 were used for all experiments.

### Cell seeding in hydrogels

The cells were suspended in 10 μl of medium and mixed with 100 μl of CMCSMA solution. Cells were suspended at a density of 1 × 10^6^ cells/ml in the precursor solution, which was then placed in 96-well plates and maintained at 37 °C and 5% CO_2_.

### Immunofluorescence staining of cell within hydrogel

To evaluate cell morphology and cytoskeletal organization, BMSCs were encapsulated in the hydrogels. Each sample (200 μl) was placed in a confocal dish, and cells were seeded at a density of 1 × 10^6^ cells/ml. Following 24 h of incubation at 37 °C under 5% CO_2_, the samples were gently washed with PBS to remove nonadherent cells. After incubation, the adherent cells were fixed in 4% paraformaldehyde for 20 min at RT and then permeabilized with 0.1% Triton X-100 in PBS for 5 min. For visualization of nuclei and actin filaments, the cells were stained using 4′,6-diamidino-2-phenylindole (DAPI) and phalloidin-iFluor 488 (Abcam, UK) as per the manufacturer’s guidelines. Fluorescent images were acquired using a confocal laser scanning microscope (LSM900w, Carl Zeiss, Germany). Both 2-dimensional (2D) and 3D z-stack reconstruction images were obtained to analyze cell morphology, spreading, and cytoskeleton structure within the hydrogel matrix. To quantify cell spreading, the surface area and perimeter of each cell were determined with ImageJ (NIH, USA). Confocal images of stained actin filaments were first converted to binary format, and individual cells were manually outlined.

### Proliferation test

The BG and BG/BMP-2 were sterilized using EO gas prior to the experiment. After EO gas sterilization, samples were stored at RT for at least 48 h to remove residual EO gas. Samples were mixed with the hydrogel at different concentrations (1%, 5%, and 10% [w/v]). The cells were then dispersed in 10 μl of medium and added to 100 μl of CMCSMA solution. The cell density was 1 × 10^6^ cells/ml. The cell-laden precursor solutions were transferred to 96-well plates and cultured at 37 °C, 5% CO_2_. Gelation was performed as previously described. Cell proliferation was assessed over 10 d using a Cell Counting Kit (CCK-8; Dojindo). The culture medium was aspirated at designated time points, and the samples were washed with PBS. Next, 100 μl of culture medium mixed with 10 μl of CCK-8 solution was added to each well. After 2 h of incubation at 37 °C, 100 μl of the supernatant from each well was transferred to a nontreated 96-well plate and the absorbance at 450 nm was measured using an ELISA Reader (Power Wave XS, BIO-TEK).

### ALP activity

Osteogenic differentiation was initially evaluated by measuring alkaline phosphatase (ALP) activity. BMSCs were seeded into hydrogels at 1 × 10^6^ within 24-well plates. After allowing 24 h for stabilization, the standard medium was replaced with an osteogenic induction medium (Stem Pro, Gibco), which was subsequently changed every 2 to 3 d. Following 7, 14, and 21 d of incubation, the hydrogel constructs were treated with 0.25% trypsin-EDTA to release the cells. The isolated cells were harvested via centrifugation and washed with PBS. Intracellular ALP activity was then quantified utilizing a commercial colorimetric ALP assay kit (ab83369, Abcam) strictly adhering to the supplier’s instructions. The final absorbance was measured at 405 nm on an ELISA reader (Power Wave XS, BIO-TEK).

### Quantitative real time-polymerase chain reaction (qRT-PCR)

BMSCs were prepared in the same manner as described in the ALP activity experiment. Quantification of osteogenic differentiation-related genes was performed using quantitative real time-polymerase chain reaction (qRT-PCR). Total RNA was extracted using TRIzol Reagent (Invitrogen, USA) and the ReliaPrep RNA Cell Miniprep System (Promega, Madison, WI, USA) following the manufacturer’s guidelines. Total RNA concentration was determined using a NanoDrop spectrophotometer (ND-1000; Thermo Fisher Scientific) and was reverse-transcribed according to the manufacturer’s protocol. Briefly, 0.1 μg of RNA was mixed with Oligo (dT) primer, 5× Reaction, RNase Inhibitor, dNTP Mix, RevertAid H Minus Reverse Transcriptase (All from Thermo Fisher Scientific), and DEPC-treated DW to a final volume of 20 μl. Reverse transcription was carried out in a thermal cycler at 42 °C for 60 min, followed by 95 °C for 5 min, and then held at 4 °C. qRT-PCR was performed using a CFX96 Touch Real-Time PCR system (Bio-Rad, USA) in 0.1-ml qPCR 8-Strip tubes (MB-q100; Gunster Biotech, New Taipei City, Taiwan) with a reaction volume of 10 μl. One microliter of each RT product was amplified in a 20-μl PCR mixture containing 300 nM of primers (Table 1) and 2× SYBR Green Supermix (Bio-Rad). Gene expression was normalized to GAPDH mRNA and calculated using the 2^−ΔΔCT^ method.

**Table 1.** Primer used for gene expression analysis

Genes	Forward primer (5′-3′)	Reverse primer (5′-3′)
*OCN*	GCA GGG TCA GGA GGA GAA TC	TTC CCT GTG TCT TAG CAG GC
*COL1*	CCG CCG CTT CAC CTA CAG C	TTT TGT ATT CAA TCA CTG TCT TGC C
*OPN*	ACA AAT ACC CAG ATG CTG TGG C	ACT TGG AAG GGT CTG TGG GG
*GAPDH*	GAT TCC ACC CAT GGC AAA TT	AAG ATG GTG ATG GGA TTT CCA TT

### Animals

Male Sprague–Dawley (SD) rats (9 to 10 weeks old) were purchased from Orient Bio (Seongnam, Republic of Korea). All animals were housed in pathogen-free rooms before the experiments.

### Surgical procedure

The experimental animals were randomly divided into 4 groups: the defect group (Defect, *n* = 5), hydrogel group (H, *n* = 5), 5% (w/v) BG-loaded hydrogel group (H/BG, *n* = 5), and 5% (w/v) BG/BMP-2-loaded hydrogel group (H/BG/BMP-2, *n* = 5).

The animals were maintained in pairs for the duration of the experiments at RT. To induce defects, SD rats were anesthetized by isoflurane inhalation. After shaving and sterilizing the back using povidone-iodine swabs, an incision was made to expose the lumbar vertebrae. A defect was created by drilling and removing the dorsal lamina of the L2 vertebra using a 2-mm micromotor handpiece (STRONG 202 N; SAESHIN, Republic of Korea). Subsequently, 100 μl of H or H/BG or H/BG/BMP-2 was injected into the defect using a 1-ml syringe (SUNGSHIM, Republic of Korea) to ensure complete filling. The fascia was repositioned manually, and the subcutaneous tissue was closed with Vicryl 6-0 sutures (ETHICON, USA). The skin was then sealed using both Vicryl 6-0 sutures. Following surgery, animals received cefazolin (33 mg/kg, twice daily) and ketoprofen (3 mg/kg, once daily) for 7 d. Eight weeks after surgery, spinal bones were removed and placed in 10% formalin at 4 °C for further analysis.

### Micro-computed tomography evaluation for spinal bone defect regeneration

For morphological observations, the specimens were examined using a desktop micro-computed tomography (CT) system (Bruker, Belgium). Scans were performed at 130 kV, 60 μA, with an exposure time of 2 min over 360°. The images were reconstructed and aligned using data viewer (Bruker, Belgium, Ver. 1.5.4.0). A cylindrical region of interest (ROI) (diameter 2 mm, height 2 mm) corresponding to the original defect site was selected from the micro-CT dataset for quantitative analysis. Bone volume fraction was calculated from the micro-CT data as the ratio of bone volume per tissue volume (BV/TV, %), trabecular thickness (Tb.Th, mm), bone mineral density (BMD, g/cm^3^), trabecular number (Tb.N, mm), bone surface (mm^3^), and bone surface density (BSD, mm^3^) using MeshLab (ISTI-CNR Research Center, Italy; Ver. 2020.03). The reconstructed images were processed in Blender (Blender Foundation, Amsterdam, Netherlands) to create 3D models.

### Histological analysis of bone repair

To evaluate bone repair histologically, samples were decalcified in Calci-Clear Rapid solution (National Diagnostics, USA) for 7 d. The specimens were then dehydrated through a graded ethanol series and embedded in paraffin. Sections of 7-μm thickness were prepared and stained with hematoxylin and eosin (H&E) and Masson’s trichrome. Also, the sections were stained with DAPI for nuclei, phalloidin conjugated with Alexa Fluor 594 (Invitrogen) for F-actin, and anti-CD31 antibodies (Abcam) to identify endothelial cells and newly formed blood vessels. The stained sections were visualized using a confocal laser scanning microscope (LSM900w, Carl Zeiss, Germany). Z-stack imaging was performed to assess vascularization and tissue organization within the regenerated defect area. To quantitatively analyze neovascularization, the fluorescence intensity of CD31-positive areas was measured. Confocal images were acquired under identical exposure settings across all groups. Mean fluorescence intensity within the defect area was quantified using ImageJ (NIH, USA) from randomly selected ROIs.

### Statistical analysis

All statistical analyses were carried out using GraphPad Prism (version 8.0), with data reported as mean ± SEM. Differences were assessed by 1-way or 2-way analysis of variance (ANOVA).

## Results

### XPS analysis of BG and BG/BMP-2

**Fig. 2.**
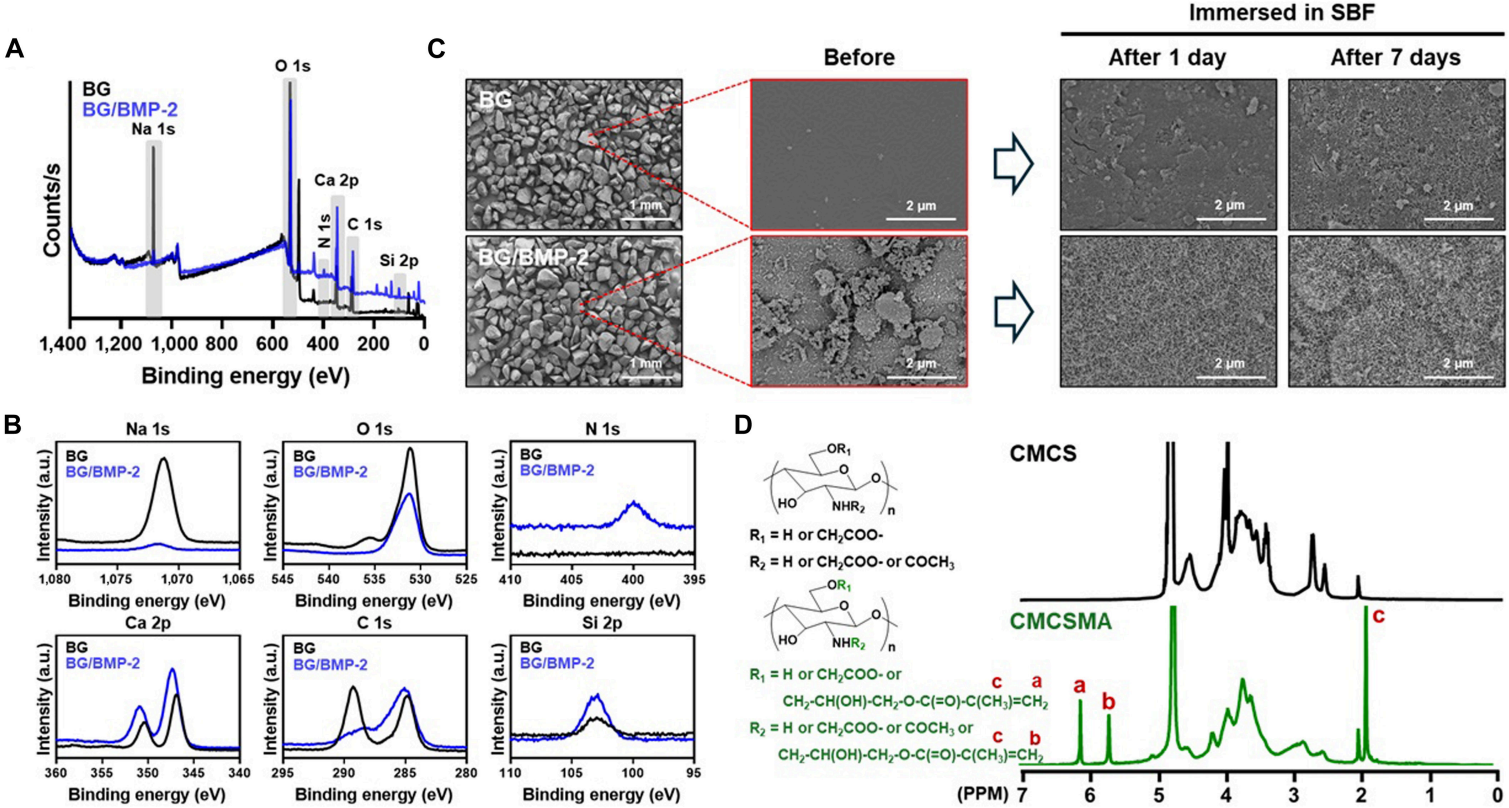
Characterization of materials. (A and B) X-ray photoelectron spectroscopy (XPS) survey spectra of BG and BG/BMP-2 composites. (C) SEM images of BG and BG/BMP-2 after being submerged in SBF solution for 7 d (operating at 2.0 kV, magnification 30× and 5,000×). Scale bars, 1 mm and 2 μm. (D) ^1^H NMR spectra of CMCS and CMCSMA.

Figure 2A and B present a comprehensive analysis of the elemental composition and binding states of BG and BG/BMP-2. Table 2 summarizes the atomic concentrations of the respective elements. Both samples exhibited characteristic peaks corresponding to O 1s (~532 eV), Si 2p (~103 eV), and Ca 2p (~347 eV), confirming the presence of typical BG elements (Fig. 2A). The narrow-scan spectra provide more detailed information regarding the chemical states of these elements (Fig. 2B). Notably, the BG/BMP-2 sample (blue line) showed an additional N 1s peak around 400 eV, attributed to nitrogen-containing groups in the BMP-2 protein. This indicates the successful immobilization of BMP-2 onto BG particles. Moreover, BG/BMP-2 exhibited a slight increase in the carbon (C 1s) peak intensity and a pronounced decrease in the sodium (Na 1s) signal from 15.89 to 1.51 compared with pure BG, which is indicative of increased organic content associated with protein coating. The pronounced decrease in Na concentration, alongside a relative increase in Ca and Si, can be attributed to the rapid dissolution behavior of the 45S5 BG in the mildly alkaline dopamine solution (pH 8.5). During the coating and subsequent washing processes, highly reactive sodium ions rapidly leached into the aqueous environment, leaving behind a silica- and calcium-rich sublayer beneath the nanometer-thick PDA coating, which was subsequently detected by XPS. XPS analysis confirms the successful coating of BMP-2 on the BG surface, while the elemental composition of the base material remains largely unchanged.

**Table 2.** Surface composition of BG and BG/BMP-2

Element	Atomic%		Element	Atomic%	
	BG	BG/BMP-2		BG	BG/BMP-2
**O 1s**	48.46	48.35	**Ca 2p**	4.84	10.39
**C 1s**	28.9	31.6	**Na 1s**	15.89	1.51
**N 1s**	0	2.83	**Si 2p**	1.9	5.32

### BMP-2 immobilization of BG and physicochemical characterization

Quantification of the BG surface immobilization efficiency of BMP-2 revealed that approximately 88% ± 2.02% of the initial BMP-2 was successfully immobilized on the BG surface (Fig. S1). The SEM images provided detailed visualization of particle morphology and size distribution (Fig. 2C). Both BG and BG/BMP-2 particles exhibited relatively uniform and similar morphologies, with no meaningful differences in shape. These observations demonstrate that coating BG particles with BMP-2 did not substantially alter their overall morphology or size characteristics. The bioactivities of BG and BG/BMP-2 were evaluated based on apatite layer formation observed in SEM images (Fig. 2C). After immersion of BG in SBF for 1 and 7 d, SEM images revealed more extensive apatite formation on the BG surface on day 7 relative to day 1. However, even on day 7, the apatite layer on BG remained thin and irregular. This limited coverage and low thickness imply that the intrinsic bioactivity of BG alone is relatively weak. In contrast, the BG/BMP-2 samples showed a more uniform and thicker apatite layer, with notably elevated bioactivity due to the BMP-2 coating (Fig. 2C). On day 1, a homogeneous, flower-like apatite layer had formed, while by day 7, multiple overlapping apatite layers were present, indicating progressive mineralization. This effect is attributed to the binding of the calcium and phosphate ions with catechol and amine moieties from PDA [22]. The formation of a denser and more homogeneous apatite layer in BG/BMP-2 implies a higher potential for bone bonding and osseointegration in comparison with pristine BG.

### Determination of methacrylation degree

Figure 2 presents the ^1^H NMR spectra of CMCS and methacrylated CMCS (CMCSMA, Fig. 2D). New peaks appearing at 5.62 and 6.07 ppm, corresponding to the vinyl protons of the methacrylate groups (–CH=CH₂), and at 1.8 ppm, attributed to the methyl protons (–CH₃), confirm the successful methacrylate modification of CMCS [23]. The DS of methacrylate groups was calculated from the integral ratio of the vinyl proton peaks (δ = 5.62 to 6.07 ppm) to the glucosamine ring protons (δ = 3.2 to 4.3 ppm) in the CMCS backbone. It was estimated to be approximately 66.7%.

### Characterization of BG or BG/BMP-2 integrated hydrogels

**Fig. 3.**
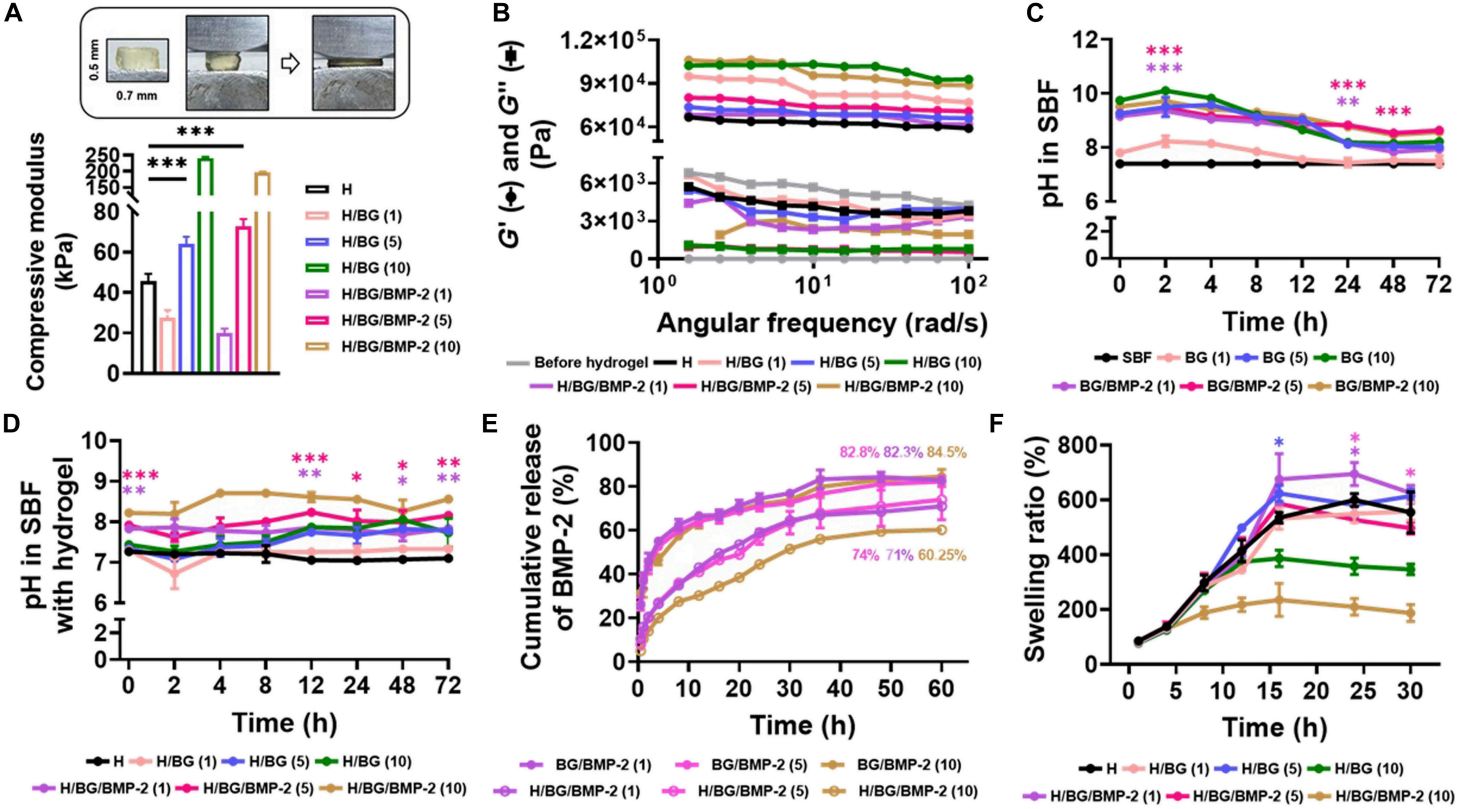
Characterization of BG and BG/BMP-2 within hydrogel. (A) The mechanical strength of hydrogels as a compressive modulus graph. (B) Frequency-dependent rheological behavior of hydrogel formulations at 1% strain. Storage (*G*′) and loss (*G*″) moduli plotted against angular frequency (rad/s). (C and D) Changes in pH of SBF solutions containing BG, BG/BMP-2, H/BG, and H/BG/BMP-2 groups. (E) BMP-2 release profiles from BG/BMP-2 groups with and without hydrogel encapsulation. (F) Swelling behavior of hydrogel samples (H, H/BG, and H/BG/BMP-2) in PBS. Data are presented as mean ± SD from 4 independent experiments (*n* = 4). Statistically significant versus control is indicated as *P < 0.05, **P < 0.01, and ***P < 0.001.

The mechanical properties were additionally examined to determine their resistance to compressive stress. All hydrogel groups withstood approximately 80% compressive strain without visible structural failure, evidencing good elasticity and durability (Fig. S2). Notably, the H/BG (10) and H/BG/BMP-2 (10) groups exhibited relatively higher compressive stress values within the 40% to 75% strain range. Moreover, H/BG (5) and H/BG/BMP-2 (5) demonstrated moderately enhanced mechanical performance compared to the control hydrogel (45 kPa), exhibiting compressive stress values in the range of 64 to 72 kPa at comparable strain levels, which suggests improved mechanical support (Fig. 3A). These data imply that hydrogels possess sufficient mechanical strength to endure physiological compressive environments, highlighting their potential suitability for in vivo applications such as bone regeneration. Rheological characterization of the hydrogel formulations was conducted using strain and frequency sweep tests. In Fig. 3B, the storage modulus (*G*′) of all hydrogel samples remained higher than the loss modulus (*G*″) across the entire frequency range (1 to 100 rad/s), indicating solid-like behavior and a stable, elastic network structure. In the strain sweep analysis, *G*′ consistently exceeded *G*″ within the linear viscoelastic region, further confirming the hydrogel’s mechanical integrity under low deformation. These rheological results confirm the excellent structural stability of the hydrogel systems, making them suitable for biomedical applications that require mechanical reliability.

To evaluate the pH stability of the materials in physiological conditions, pH was monitored over 72 h (Fig. 3C and D). The groups without hydrogel initially exhibited a slight increase in pH, followed by a gradual decrease over time, whereas the hydrogel-containing groups (H/BG and H/BG/BMP-2) maintained relatively stable pH levels throughout the immersion period. The BG and BG/BMP-2 groups consistently maintained an alkaline environment with a pH above 8 (Fig. 3C). Conversely, the hydrogel composites (H/BG and H/BG/BMP-2) maintained physiological pH levels, although the pH of the highest BMP-2 concentration group [H/BG/BMP-2 (10)] increased (Fig. 3D). By moderating alkalinity, incorporating BG or BG/BMP-2 into the hydrogel matrix more closely reproduces physiological conditions than the group without the hydrogel matrix, thereby creating a more stable pH environment favorable for cell activity and osteogenesis [3,18,24].

BMP-2 release from the BG/BMP-2 and H/BG/BMP-2 groups was quantified using an ELISA kit. In the BG/BMP-2 group, a rapid initial release of 57% to 62% of the total BMP-2 was observed within the first 8 h, followed by a cumulative release of 82% to 84% at 60 h. In contrast, the H/BG/BMP-2 group exhibited a markedly reduced initial burst release of 27% to 36% within the first 8 h, with a cumulative release of 60% to 74% after 60 h (Fig. 3E). These results suggest that encapsulation of BG/BMP-2 within the hydrogel matrix effectively delays BMP-2 release, resulting in a more sustained release profile.

The swelling behaviors of the hydrogels were investigated by immersing the hydrogel samples in PBS at physiological temperature. The hydrogel samples were weighed at different time points to determine the degree of swelling. Most hydrogels exhibited a rapid initial swelling phase, followed by stabilization as equilibrium was reached (Fig. 3F). The rate and extent of swelling were inversely related to the concentrations of BG and BG/BMP-2 in the precursor solution, with higher concentrations resulting in lower equilibrium swelling ratios and more rigid hydrogel structures. These results show that the concentration of BG and BG/BMP-2 affects the swelling behavior of the hydrogel.

### Cell morphology encapsulated within hydrogel

**Fig. 4.**
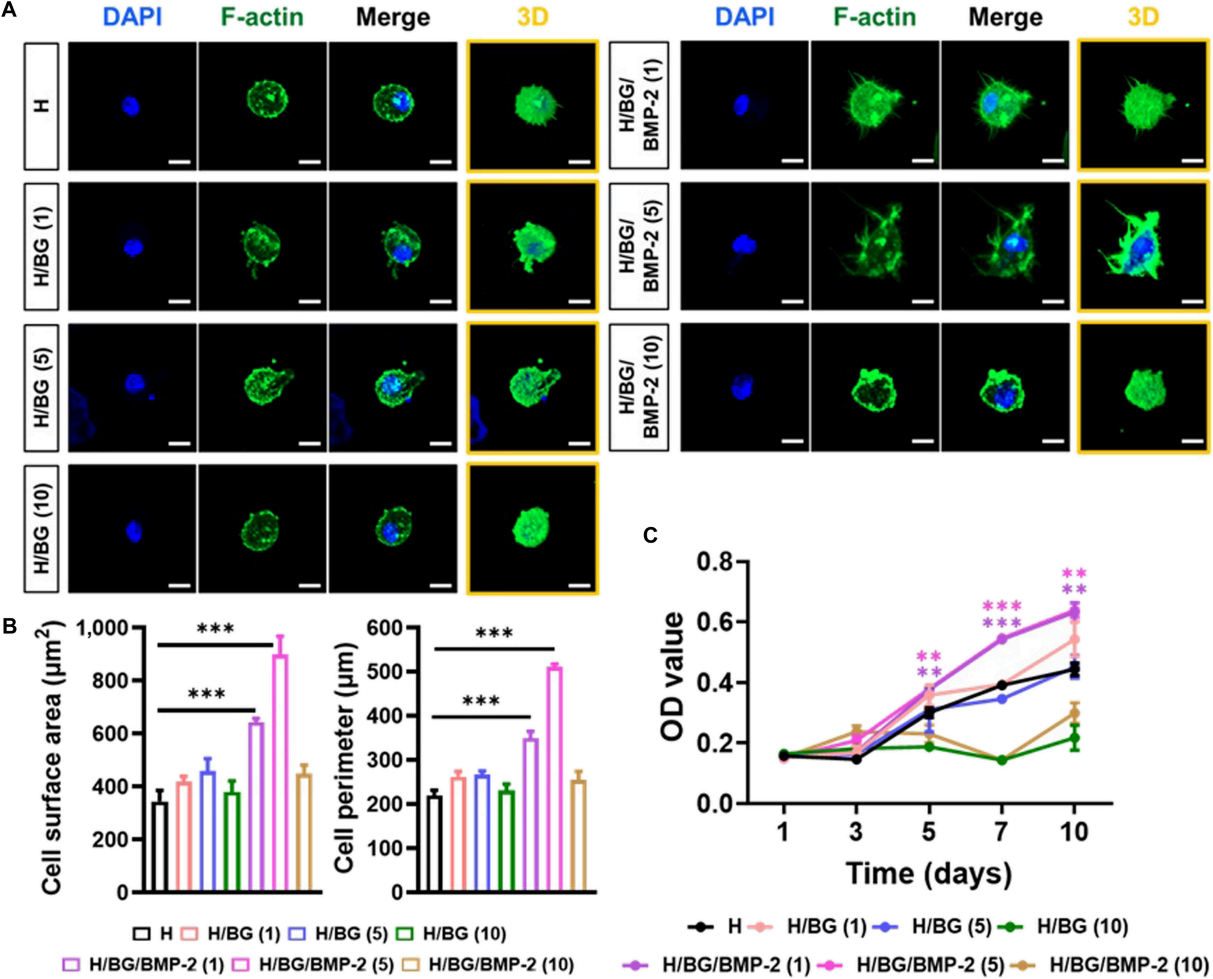
Analysis of cellular changes within hydrogel environments. (A) Immunofluorescence staining images of BMSC encapsulated in hydrogels containing BG and BG/BMP-2 after 24 h of culture (Nuclei, blue; F-actin, green). Scale bars, 10 μm. (B) Surface area and perimeter of BMSCs encapsulated in the hydrogels. (C) Proliferation of cultured BMSCs in hydrogel composites over 10 d. Data are presented as mean ± SD from 4 independent experiments (*n* = 4). Statistical comparisons to controls are indicated by **P < 0.01 and ***P < 0.001.

To investigate the morphology of BMSCs encapsulated within the hydrogel, we performed immunofluorescence staining of nuclei (DAPI, blue) and F-actin (phalloidin, green), followed by 3D confocal imaging. Representative images revealed distinct morphological characteristics depending on the hydrogel composition (Fig. 4A). Cells encapsulated in the hydrogel exhibited a rounded morphology with compact actin organization. Similarly, in BG-containing hydrogels (H/BG groups), BMSCs displayed limited spreading and maintained a predominantly spherical shape. In contrast, cells cultured in hydrogels incorporating BG/BMP-2 (H/BG/BMP-2 groups) exhibited extended filopodia and a well-spread cytoskeleton, which promoted cell–matrix interactions. Remarkably, cells in the H/BG/BMP-2 (1) or (5) exhibited more pronounced spreading and cytoskeletal organization than those in the H/BG groups, suggesting that BMP-2 further facilitates cell spreading and attachment within the hydrogel matrix. The 3D reconstructions (highlighted in yellow boxes) further confirmed these morphological differences, particularly regarding actin fiber extension and spatial distribution. Quantitative analysis of cell area and circularity revealed notably increased spreading and decreased circularity in the BG/BMP-2-containing hydrogels, consistent with cytoskeletal remodeling (Fig. 4B). These findings imply that the BG or BG/BMP-2 incorporation plays a key role in modulating cellular behavior and morphology within 3D hydrogel environments.

### BMSC proliferation within hydrogels incorporating BG and BG/BMP-2

The proliferation rates of cells treated with various concentrations of BG and BG/BMP-2 were evaluated (Fig. 4C). The data suggest that cell proliferation rates varied depending on the concentration of BG or BG/BMP-2. In most groups, the proliferation rate gradually increased throughout the culture period. However, in the H/BG (10) and H/BG/BMP-2 (10) groups, a reduction in cell proliferation was observed over time, suggesting that the highest concentration may adversely affect cellular growth. Interestingly, after an initial decline, a slight increase in proliferation was noted on day 10, demonstrating a potentially delayed cellular adaptation to the dense matrix environment. These results demonstrate that the concentration of BG or BG/BMP-2 within the hydrogel matrix is critical for modulating cell proliferation. At elevated BG and BG/BMP-2 concentrations, the denser network may restrict nutrient and oxygen diffusion, creating a microenvironment less conducive to cell growth [25].

### Osteogenic differentiation of BMSCs within BG and BG/BMP-2 hydrogel

**Fig. 5.**
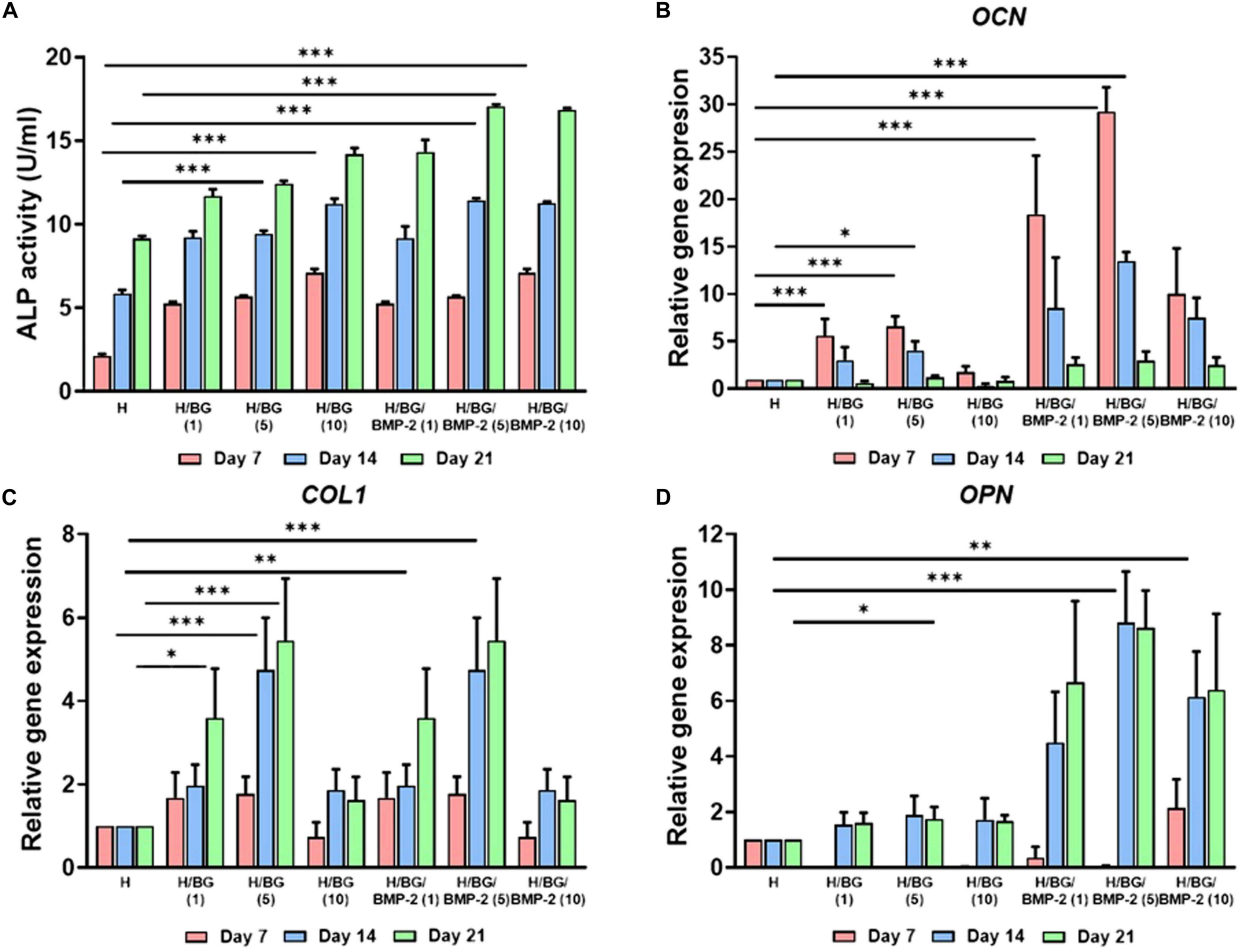
In vitro evaluation of osteogenic differentiation within H/BG and H/BG/BMP-2 composites at different concentrations. (A) Alkaline phosphatase (ALP) activity assay for quantitative assessment of osteogenic differentiation in BMSCs. (B to D) Relative gene expression levels of *OCN*, *COL1*, and *OPN* to evaluate the osteogenic differentiation of BMSCs. Data are presented as mean ± SD from 4 independent experiments (*n* = 4). Significant differences compared to controls are denoted by *P < 0.05, **P < 0.01, and ***P < 0.001.

ALP activity was measured to assess the osteogenic differentiation potential of the samples at 7, 14, and 21 d across different BG and BG/BMP-2 concentrations. ALP activity increased in a time-dependent manner, indicating active osteogenic differentiation during the incubation period (Fig. 5A). BMSCs cultured in the BG/BMP-2-containing CMCSMA hydrogel exhibited notably higher ALP activity than those cultured in hydrogel-only or BG-containing groups. Among these, the BG/BMP-2 (5) group showed the highest values, reaching 11.44 ± 0.10 and 17.06 ± 0.10 on days 14 and 21, respectively. These results highlight the gradual release of BMP-2 coated on BG, which subsequently induced ALP activity in BMSCs. RT-PCR was conducted to observe the effects of the hydrogel formulations on the osteogenic differentiation of BMSCs. The gene expressions of *OCN* (Fig. 5B), *COL1* (Fig. 5C), and *OPN* (Fig. 5D) were up-regulated in cells cultured within CMCSMA hydrogel containing BG and BG/BMP-2 relative to the control group (cultured in CMCSMA hydrogel alone). Considerably high *OCN* expression was observed for the hydrogel containing BG/BMP-2 compared to the other groups on days 7 and 14. Increased expression of *COL1*, a structural component of the extracellular matrix, was observed in all groups on days 14 and 21. In particular, the expression of the *OPN* gene, which regulates bone matrix formation, was up-regulated in the BG/BMP-2 (5) group (8.62 ± 1.17) relative to the control group on day 21. These results indicate that BG/BMP-2 within the hydrogel formulations positively influenced the osteogenic differentiation of BMSCs.

### Micro-CT analysis for rat spinal bone regeneration

**Fig. 6.**
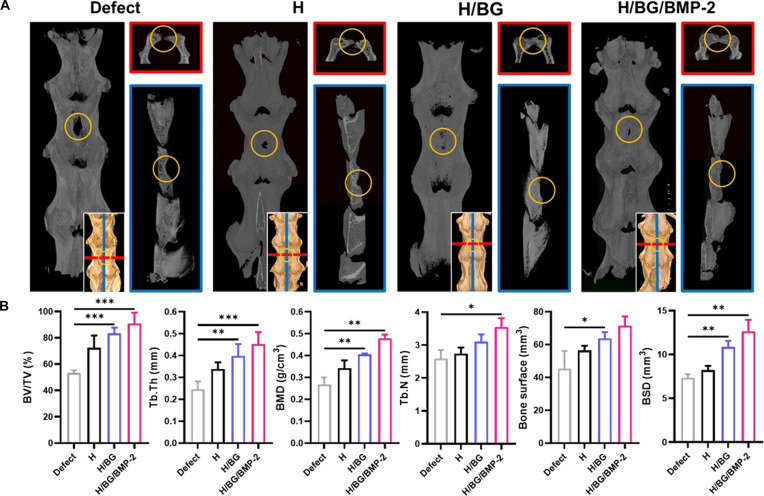
Micro-CT analysis of spinal bone defect regeneration in vivo. (A) Dorsal view of the 3D reconstruction and coronal cross-sections from micro-CT scans through the center of the rat spinal bone defect. (B) Quantitative evaluations of BV/TV, Tb.Th, BMD, Tb.N, bone surface, and BSD within the defect area. Data are presented as mean ± SD of 3 independent experiments (*n* = 5). Statistical comparisons to controls are indicated by *P < 0.05, **P < 0.01, and ***P < 0.001.

The H/BG/BMP-2 group, specifically H/BG/BMP-2 (5), was selected for further evaluation as it showed improved cell proliferation and strong up-regulation of osteogenic markers in vitro. We subsequently performed in vivo analyses using a rat spinal defect model, examining 4 experimental groups: Defect, H, H/BG, and H/BG/BMP-2. Figure 6 presents the micro-CT imaging conducted 8 weeks after implantation to assess the bone regenerative capacity of the samples. In the control group (Defect), new bone formation was minimal, as evident in the reconstructed 3D image (yellow circle). The defect remained largely unfilled, suggesting limited endogenous bone regeneration under these conditions (Fig. 6A). In contrast, both the hydrogel-only and the hydrogel with BG or BG/BMP-2 groups presented bone bridging and mineral deposition at the defect site. The micro-CT cross-sectional images (highlighted boxes in red for the axial plane and blue for the sagittal plane) in the H/BG/BMP-2 group reveal a denser and more continuous bone structure related to the hydrogel-only group. Quantitative analysis confirmed a considerable increase in BV/TV, Tb.Th, BMD, Tb.N, bone surface, and BSD in the H/BG/BMP-2 group in comparison with the Defect, H, and H/BG groups, implying that BG/BMP-2 effectively induced osteoinduction (Fig. 6B).

### Histological analysis

**Fig. 7.**
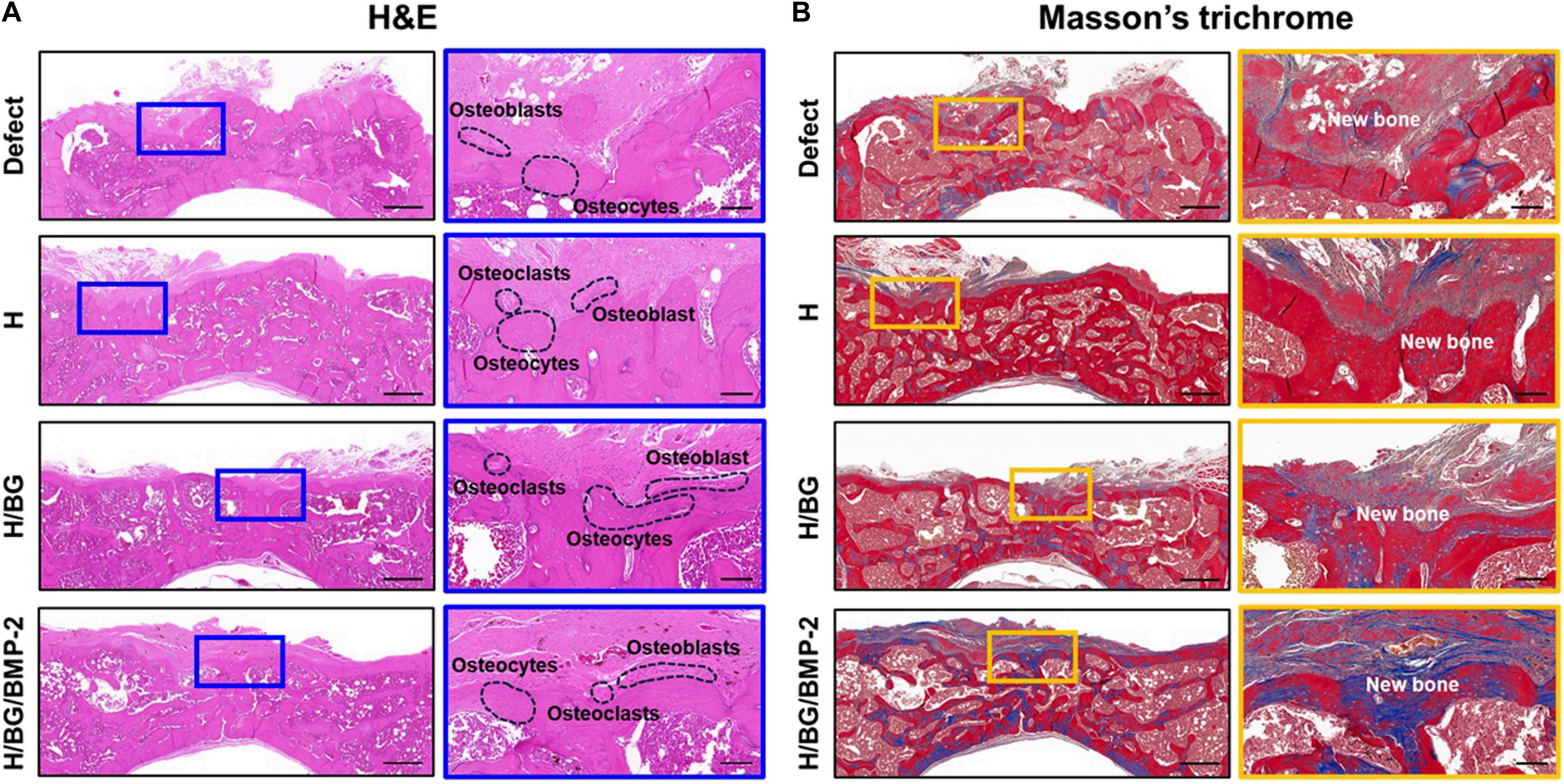
Histological evaluation of spinal bone defect regeneration. (A) Hematoxylin and eosin (H&E) staining and (B) Masson’s trichrome staining images exhibited the integration of new bones in between defect areas after treatment of H, H/BG, and H/BG/BMP-2 at 8 weeks post-implantation. Scale bar, 500 and 100 μm.

Bone regeneration capacity was assessed through histological analysis**.** Eight weeks after implantation, distinct differences in cellular activity among the groups were identified by H&E staining (Fig. 7A). In the experimental group treated with H/BG/BMP-2, a high density of osteoblasts was observed along the newly formed bone matrix, reflecting active bone formation. The presence of osteocytes embedded in the bone matrix demonstrated successful maturation of the newly formed bone [26,27]. Interestingly, osteoclasts were detected near remodeling sites, confirming ongoing bone turnover, a sign of balanced remodeling essential for stable bone regeneration. Residual hydrogels were also found in some regions, showing gradual integration with the surrounding tissue. Masson’s trichrome staining provides additional indication of facilitated bone tissue regeneration in the H/BG/BMP-2 group. New bone formation was visualized by red staining, evidencing collagen deposition and mineralization (Fig. 7B). Fibrous collagen, representing the formation of the bone matrix, was stained blue. This suggests that a well-organized bone structure is formed through the assembly of mineralized collagen fibers. Compared to the control group (defect), where minimal new bone was observed, the experimental groups revealed a well-organized bone structure with extensive matrix formation.

**Fig. 8.**
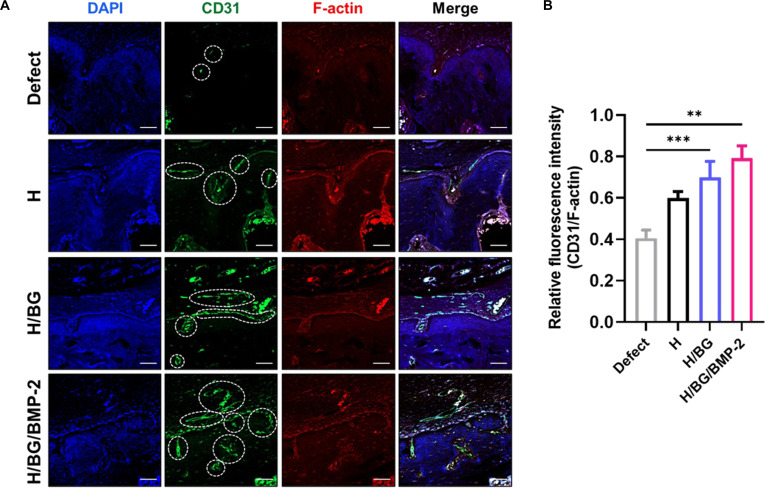
Immunofluorescence staining analysis of neovascularization in spinal bone defect areas. (A) Immunofluorescence images depicting CD31 expressions in the defect areas. White circles denote newly formed blood vessels. (B) Quantification of CD31 fluorescence intensity based on the confocal images shown in (A). Statistical significance versus controls is indicated by **P < 0.01 and ***P < 0.001. Scale bar, 50 μm.

Additionally, to assess angiogenesis, a key indicator of bone regeneration, immunofluorescence staining was performed in a rat spinal defect model using CD31 (green) as an endothelial cell marker, F-actin (red), and DAPI (blue) to visualize cell nuclei. In Fig. 8, CD31-positive signals were sparsely observed in the control group (Defect), reflecting limited vascular formation. Conversely, the H, H/BG, and H/BG/BMP-2 groups exhibited increased CD31 expression, with the H/BG/BMP-2 group showing a higher density of CD31-positive regions (highlighted by white dashed circles), reflecting developed neovascularization. The merged images revealed that the newly formed vessels were more integrated within the regenerating tissue in the H/BG/BMP-2 group, supporting the role of BMP-2 in promoting vascularized bone healing. Quantitative analysis of the CD31-positive area confirmed a statistically significant increase in the H/BG/BMP-2 group as compared with the other groups, highlighting the synergistic effect of BG and BMP-2 in facilitating vascularized bone regeneration.

## Discussion

In this study, we investigated the effects of incorporating BMP-2-coated BG into a light-curable CMCSMA hydrogel on spinal bone regeneration. Our findings demonstrated that BMP-2 coating to BG substantially enhanced cellular behavior in vitro and further confirmed its bone regeneration potential in vivo experiments [28–30]. The CMCSMA hydrogel matrix functions as a light-curable system that provides an effective platform for the dual delivery of BG and BMP-2 [31,32].

The first aspect to be discussed in this study is the use of a PDA layer to coat BMP-2 onto BG. Dopamine polymerizes into PDA, which provides catechol/amine groups that bind BMP-2 through covalent and noncovalent interactions. These interactions enable BMP-2 to be stably anchored to the BG surface [29,33]. Another advantage of employing dopamine is that PDA coatings can promote biomimetic apatite mineralization when exposed to SBF. The catechol and amine groups of PDA act as nucleation sites for these ions, thereby promoting apatite deposition on BG (Fig. 2A to C) [33,34].

Hydrogel may offer structural support to overcome the inherent limitations of BMP-2-coated BG particles, thereby maintaining mechanical stability and accelerating regenerative processes. In this regard, CMCS was selected and subsequently methacrylated (Fig. 2D). Methacrylation introduces reactive methacrylate groups that enable photo crosslinking, thereby converting CMCS from a soluble polymer into a stable hydrogel network with tunable mechanical and biological properties [35]. The increase in the mechanical strength of the hydrogel can be primarily attributed to the incorporation of BG particles within the crosslinked CMCSMA matrix. The rigid inorganic particles act as reinforcing fillers, restricting polymer chain mobility and thereby enhancing the load-bearing capacity of the hydrogel. In addition, the strong interfacial interactions between the BG and the hydrogel network facilitate efficient stress transfer, which further contributes to the overall mechanical stability. As a result, the composite hydrogel exhibits superior mechanical performance compared to the pristine CMCSMA hydrogel (Fig. 3A and B) [35]. While the compressive modulus of the optimized hydrogel (64 to 72 kPa) is lower than that of native load-bearing trabecular bone (typically 1 to 10 MPa), it is important to emphasize that this system is designed as an injectable, space-filling matrix rather than a primary load-bearing implant. In clinical scenarios, primary mechanical stabilization of spinal defects is achieved via rigid internal fixation devices. The hydrogel provides a sufficient mechanobiological niche to withstand local physiological strains, prevent material wash-out, and seamlessly adapt to irregular defect geometries to support cellular infiltration.

When BG/BMP-2 particles are directly exposed to the culture medium, the rapid dissolution of cations such as Ca^2+^ and Na^+^ can lead to an increase in the local pH, which may influence the cellular microenvironment. However, embedding BG/BMP-2 within the hydrogel (H/BG/BMP-2) moderates the release of ions. The PDA layer on the BG particles acts as a physical diffusion barrier that effectively mitigates the initial rapid burst release of alkaline ions. This controlled ion release, combined with the buffering capacity of the hydrogel network, attenuates the abrupt rise in pH and creates a more stable, physiological microenvironment (Fig. 3C and D). In addition to this buffering effect, the hydrogel also plays a crucial role in regulating BMP-2 release. While PDA coating on BG particles already provides a controlled release mechanism by immobilizing BMP-2 through covalent and noncovalent interactions, the hydrogel matrix introduces an additional diffusion barrier [29,33]. This dual regulation, PDA immobilization combined with hydrogel encapsulation, results in a more sustained and predictable release profile of BMP-2 (Fig. 3E) [28–30,33]. Taken together, the hydrogel not only mitigates the pH increase associated with BG dissolution but also enhances the sustained release of BMP-2 beyond the capacity of PDA coating alone. This synergistic effect highlights H/BG/BMP-2 as a promising composite system for bone tissue engineering, offering both improved bioactivity and a more favorable microenvironment for cellular responses [28–30,33]. Furthermore, the sustained release of BMP-2 over the initial 60 to 72 h serves as a potent biological trigger for the osteogenic cascade. This short-term release is biologically sufficient to recruit endogenous mesenchymal stem cells and osteoprogenitors to the defect site. Following this initial trigger, the recruited cells mediate long-term bone regeneration up to 8 weeks via autocrine and paracrine signaling and active extracellular matrix deposition.

Compared to the pristine hydrogel (H), both H/BG and H/BG/BMP-2 composites promoted enhanced adhesion and spreading of BMSCs, with H/BG/BMP-2 showing the most pronounced effect (Fig. 4). This improvement can be explained by several combined mechanisms. First, the incorporation of BG facilitated the formation of amorphous calcium phosphate (ACP) on the particle surface, which is known to promote focal adhesion assembly and strengthen cell–matrix interactions [31–34]. Second, in H/BG/BMP-2, the presence of BMP-2 immobilized via PDA coating further enhanced ACP formation (Fig. 2C), thereby amplifying the osteoconductive microenvironment [29,30]. Third, BMP-2 signaling itself, through Smad-dependent and noncanonical pathways, may indirectly influence focal adhesion dynamics via crosstalk with integrin-mediated signaling [35]. Taken together, these factors explain why H/BG/BMP-2 exhibited superior performance in supporting BMSCs adhesion and spreading compared to H/BG alone. The results highlight the importance of combining bioactive inorganic phases with growth factor delivery systems to achieve both biochemical and biophysical cues that interactively promote osteogenic cell responses.

The enhanced osteogenic differentiation observed in BMSCs cultured within BG/BMP-2-containing hydrogels can be attributed to the combined effects of both BMP-2 and silicate ions released from BG (Fig. 5). BMP-2, as a protein growth factor, exerts its influence through direct ligand–receptor interactions with BMP receptors, activating canonical Smad signaling cascades and thereby inducing precise transcriptional regulation of osteogenic genes [36]. This direct signaling mechanism explains the strong up-regulation of ALP activity and osteogenic markers such as *OCN*, *COL1*, and *OPN* in the BG/BMP-2 groups [29,30]. In parallel, silicate ions released from BG contribute to osteogenesis through an indirect mode of action. Unlike BMP-2, silicate ions lack dedicated receptors; instead, they alter the extracellular environment by modulating pH and ionic balance, which in turn activates intracellular signaling pathways such as MAPK and Wnt/β-catenin [37,38]. These environmental stimuli drive cells to up-regulate bone-related genes and proteins, thereby supporting both proliferation and differentiation in a less specific but complementary manner. The combination of these 2 mechanisms, precise, receptor-mediated signaling from BMP-2 and supportive, environment, modulating effects from silicate ions, provides a dual stimulus for osteogenesis. BMP-2 delivers targeted osteoinductive signals, while silicate ions act as modulators that enhance the cellular microenvironment and reinforce osteogenic gene expression. This synergy explains why H/BG/BMP-2 exhibited superior outcomes compared to H/BG alone, with enhanced adhesion, spreading, and differentiation capacity of BMSCs. Collectively, these findings highlight the importance of integrating both biochemical (BMP-2) and biophysical/ionic (silicate ions) inputs within hydrogel scaffolds to achieve optimal bone tissue engineering outcomes.

The in vivo analyses demonstrated that the H/BG/BMP-2 scaffold promoted not only bone formation but also balanced remodeling and vascularization (Figs. 6 to 8). Micro-CT revealed dense bone bridging with improved structural parameters, while histological staining confirmed active osteoblast activity, embedded osteocytes indicating maturation, and osteoclasts at remodeling sites. These findings suggest that the regenerated bone was physiologically active and undergoing turnover, a prerequisite for long-term stability [39,40]. Masson’s trichrome staining highlighted organized mineralized collagen fibers, evidencing robust matrix deposition. Furthermore, immunofluorescence staining for CD31 showed enhanced neovascularization in the H/BG/BMP-2 group, indicating that BMP-2 contributed to vascularized bone healing [36,39]. Importantly, these in vivo findings are consistent with the in vitro results, where H/BG/BMP-2 scaffolds promoted cell adhesion, proliferation, and strong up-regulation of osteogenic markers [29,30]. The enhanced osteogenic differentiation observed in vitro was reflected in vivo by the formation of mature bone tissue and the establishment of vascularized structures. This correlation highlights the translational relevance of the dual mechanism, direct BMP-2 signaling and indirect ionic modulation by BG, in driving both cellular differentiation and functional bone regeneration [36,39,40]. The in vivo evaluation was conducted over an 8-week period to specifically assess the terminal remodeling and maturation of the newly formed bone, while the early osteogenic potential was extensively confirmed by our in vitro assays. Moreover, the safety of the highly swellable hydrogel (swelling ratio > 500%) within the confined spinal space was indirectly validated. To prevent neural compression, the defects were slightly underfilled (approximately 70% to 80% of the defect volume). Throughout the 8-week observation period, no animals exhibited neurological deficits, such as hind limb paralysis, indicating that this underfilling strategy successfully accommodated the material’s swelling without compromising the spinal cord.

Despite these promising results, several limitations remain that should be addressed in future studies. Extended in vivo experiments are necessary to confirm whether the newly formed bone achieves long-term structural integrity and functions effectively under dynamic loading conditions [39,40]. Additionally, optimizing BMP-2 release kinetics to ensure precise bioavailability, along with improving the mechanical properties and biocompatibility of the hydrogel, is essential to enhance its potential for clinical applications [35].

## Conclusion

Overall, this study reflected that BMP-2-coated BG incorporated into a light-curable CMCSMA hydrogel effectively induces osteogenesis and provides a controlled BMP-2 delivery platform [28–32]. This approach represents a promising biomaterial-based strategy for spinal bone regeneration, warranting further investigation into its clinical applications [39,40].

The present study demonstrated the efficacy of incorporating BMP-2-immobilized BG (BG/BMP-2) into a light-curable chitosan-based hydrogel for spinal bone defect repair. By immobilizing BMP-2 through a PDA coating, we achieved sustained growth factor release while harnessing the notable bioactive ion dissolution of BG to enhance osteogenesis. Our in vitro experiments showed concentration-dependent BMP-2 binding, improved cell proliferation, and strong up-regulation of osteogenic markers (*ALP*, *OCN*, *COL1*, and *OPN*) at moderate BG/BMP-2 loads. In vivo validation using a rat spinal defect model further confirmed substantial new bone formation, increased bone volume fraction, and organized lamellar structures in the H/BG/BMP-2-treated groups. Importantly, the photocurable CMCSMA hydrogel facilitated minimally invasive applications by enabling close adaptation to irregular spinal defects. These findings highlight the synergistic benefits of combining BG-derived ionic stimulation, localized BMP-2 activity, and practical hydrogel handling within a single platform.

## Ethical Approval

The animal experimental procedures were approved by the Institutional Animal Care and Use Committee (IACUC) of the School of Medicine, Catholic University of Korea (CUMC-2023-0094-01). All animal procedures were conducted in accordance with the Animal Protection Act and the Guide for the Care and Use of Laboratory Animals, as approved by the IACUC of the Catholic University of Korea.

## Data Availability

Data will be made available on request.
