# Supplementary material for: Light-Curable Methacrylated Carboxymethyl Chitosan Hydrogel Incorporating Bone Morphogenic Protein-2-Immobilized Bioactive Glass for Spinal Bone Regeneration
Source: Biomater Res. 2026 Jul 7;30:0391. doi: 10.34133/bmr.0391 (PMC13338563; doi:10.34133/bmr.0391)
Supplement: Supplementary 1 — Figs. S1 and S2 [file bmr.0391.f1.docx]

**Supplementary information**

**Light Curable Methacrylated Carboxymethyl Chitosan Hydrogel Incorporating Bone Morphogenic Protein-2–Immobilized Bioactive Glass for Spinal Bone Regeneration**

Mi Yeon Ha^a,1^, Gun-Jae Jeong^b,1^, Jae Taek Hong^c,1^, Hye June Byun^a,d^, Dae Hyeok Yang^a^, Ju Woong Jang^e^, and Heung Jae Chun^a,c,f *^

^a^*Institute of Cell and Tissue Engineering, College of Medicine, The Catholic University of Korea, Seoul 06591, Republic of Korea*

^b^*School of Nanomedical Engineering, Korea National University of Transportation, Chungcheongbuk-do 27469, Republic of Korea*

^c^*Department of Neurosurgery, Eunpyeong St. Mary’s Hospital, College of Medicine, The Catholic University of Korea, Seoul 03312, Republic of Korea*

^d^*Department of Medical Sciences, College of Medicine, The Catholic University of Korea, Seoul 06591, Republic of Korea*

^e^*Renew Medical Co. Ltd. Jeongju-ro, Bucheon-si, Gyeonggi-do 14532, Republic of Korea*

^f^*Department of Medical Life Sciences, College of Medicine, The Catholic University of Korea, Seoul 06591, Republic of Korea*

^1^These authors have equal contribution.

*** Corresponding author. Heung Jae Chun**

Institute of Cell and Tissue Engineering, College of Medicine, The Catholic University of Korea, 222, Banpo-daero, Seocho-gu, Seoul, 06591, Republic of Korea

Tel: +82 2 3147 8403, Email: [chunhj@catholic.ac.kr](mailto:chunhj@catholic.ac.kr)


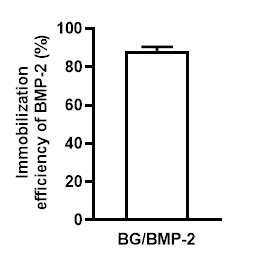


**Figure S1.** Immobilization efficiency of BMP-2


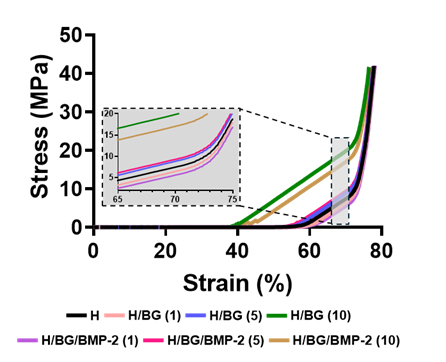


**Figure S2.** Compressive stress-strain profiles of the hydrogel composites.
